# Therapeutic Outcomes of Graduated and Progressive Compression in Chronic Venous Disorders: A Meta-Analysis

**DOI:** 10.7759/cureus.96146

**Published:** 2025-11-05

**Authors:** Hosam Hadi Hassan Awaji, Ibrahim Adil Hamadelniel Alhadi, Albasil Hamoud E Badawi, Faisal Ali Manea, Raghad M Altwiraqi, Fahad Mohsin Alotaibi, Ahmed M Mohamed, Shouq Naif Aloufi, Zahraa S Alsultan, Maha Abbas Alotaibi, Abdullah Alhajraf

**Affiliations:** 1 Department of Preventive Medicine, North West Armed Forces Hospital, Tabuk, SAU; 2 Department of General Surgery, Royal College of Surgeons of Edinburgh, Edinburgh, GBR; 3 Department of Human Anatomy, University of Gezira, Wad Madani, SDN; 4 Department of General Surgery, University of Gezira, Wad Madani, SDN; 5 Faculty of Medicine, King Abdulaziz University, Jeddah, SAU; 6 Faculty of Medicine, Najran University, Najran, SAU; 7 Department of Medicine, College of Medicine, Taif University, Taif, SAU; 8 Faculty of Medicine, Imam Muhammad Ibn Saud Islamic University, Riyadh, SAU; 9 Department of Trauma, University of Gezira, Wad Madani, SDN; 10 Faculty of Medicine, University of Taibah, Medina, SAU; 11 Department of Medicine, Alfaisal University College of Medicine, Riyadh, SAU; 12 Faculty of Medicine, Qassim University, Qassim, SAU; 13 Faculty of Medicine, Royal College of Surgeons in Ireland, Dublin, IRL

**Keywords:** chronic venous diseases, elastic compression stockings, graduated, progressive, rcts

## Abstract

Chronic venous disorders (CVD) are a common health problem affecting millions of people worldwide. Compression therapy, including graduated compression (graduated elastic compression stockings (GECS)) and progressive compression (progressive elastic compression stockings (PECS)), is a widely used treatment for CVD. This meta-analysis aimed to determine whether PECS provides superior clinical and hemodynamic outcomes compared to GECS in patients with CVDs. Clinical efficacy was defined as the disappearance or significant improvement in the sensation of fatigue or heaviness as reported by patients or assessed through validated symptom scales. A systematic literature search was conducted in PubMed, EMBASE, and Cochrane databases to identify randomized controlled trials (RCTs) comparing GECS and PECS for CVD from inception to November 23, 2024. The primary outcomes were clinical improvement, interface pressure, and ejection fraction. Secondary outcomes included tightness, ease of donning, and adverse events. Data were pooled using a random-effects meta-analysis model, with odds ratios (ORs) and mean differences (MDs) calculated along with 95% confidence intervals (CIs). Five RCTs involving 874 patients were included in the meta-analysis. PECS demonstrated significantly higher clinical improvement rates compared to GECS. Additionally, PECS resulted in a significantly higher interface pressure at level C (calf level), which is crucial for optimal venous hemodynamics. However, no significant difference was found between the two modalities in terms of improving ejection fraction. Secondary outcomes, such as tightness and ease of donning, showed no significant differences between the two groups. This meta-analysis suggests that PECS may be a more effective treatment option for CVD compared to GECS. However, further high-quality RCTs with longer follow-up periods are needed to confirm these findings and to assess the long-term benefits of PECS.

## Introduction and background

Chronic venous disorders (CVDs) represent a spectrum of venous system pathology, ranging from benign telangiectasias and varicose veins to severe diseases such as edema, skin changes, and venous ulcers, all of which significantly impact the quality of life of patients. Compression therapy is a cornerstone in the management of CVD, with the aims of improving venous return, reducing venous pressure, and reducing symptoms at every level of disease. It is most commonly used in patients with chronic venous insufficiency (CVI), varicose veins, and even active or healed venous ulcers, if arterial insufficiency is excluded [1].

Among the available options, graduated elastic compression stockings (GECS) and progressive elastic compression stockings (PECS) are the most widely utilized [1]. GECS involve a decreasing pressure gradient distally to proximally through the limb segments to counteract hydrostatic pressure and enhance venous return. PECS provides a more constant pressure through the limb, with venous support equal throughout, which has the potential to decrease venous pooling and hypertension, which can be beneficial in patients with more severe disease [2].

PECS are also simpler to implement and are better tolerated, which can increase patient compliance, a strong predictor of long-term results. Enhanced compliance can maximize symptom reduction and minimize recurrence rates for venous ulcers [3].

Despite widespread use, the relative efficacy of GECS and PECS remains debated. While GECS is traditionally preferred and widely used, PECS offers a plausible alternative, especially in cases where GECS is not well-tolerated or contraindicated [4]. Some evidence suggests that PECS may be as effective as GECS in reducing symptoms and preventing the progression of CVD, but due to the few head-to-head randomized trials that have compared their hemodynamic outcomes, and existing studies are limited by small sample sizes and short follow-up durations, further research is needed to establish its role definitively [5].

Therefore, we performed a systematic review and meta-analysis to directly compare the clinical and hemodynamic efficacy of GECS and PECS in patients with CVD, aiming to resolve inconsistencies in prior evidence.

## Review

Methods

The conduction and reporting of this meta-analysis followed the principles of the Cochrane Handbook for Systematic Reviews of Interventions, version 6, and the Preferred Reporting Items for Systematic Reviews and Meta-Analyses (PRISMA) guidelines [6].

The research question

In patients with CVDs, what is the comparative efficacy of graduated compression (GC) versus progressive compression (PC) in terms of clinical outcomes?

Research aims and objectives

Research Aims

This research aims to compare the efficacy of GC and PC in the management of CVDs, to provide a comprehensive evaluation of the existing evidence on the use of GC and PC in CVD, and to offer insights into the most effective compression strategy for the management of CVD.

Research Objectives

The objectives of this research are to conduct a systematic review of the literature on GC and PC in CVD, to perform a meta-analysis of studies comparing the clinical outcomes of GC and PC in CVD, to assess the impact of GC and PC on symptom relief, disease progression, and recurrence of venous ulcers in patients with CVD, to evaluate patient compliance with GC and PC therapies, and to identify gaps in the current evidence and suggest directions for future research.

Search strategy

Electronic Searches

The following electronic databases were searched for eligible studies: PubMed, EMBASE, Cochrane Central Register of Controlled Trials (CENTRAL), Web of Science, ProQuest, and Scopus. The search was set for all articles published in English from inception until November 23, 2024.

The following search terms were used: (chronic venous insufficiency) AND (graduated compression stockings OR GECS) AND (progressive compression stockings OR negative compression stockings OR degressive compression stockings OR PECS). We used no filters by language or publication period. Reference lists of retrieved papers were also manually screened to ensure inclusion of all relevant studies, and preprints were evaluated for potential inclusion if they contained sufficient methodological detail.

Eligibility Criteria

Inclusion: Randomized controlled trials (RCTs) and controlled comparative studies evaluating GECS versus PECS in adults with CVD. Exclusion: Animal studies, retrospective designs, reviews, case series (<4 patients), conference abstracts, and duplicates.

Appendix A summarizes the search terms used for each database and the count of search results. The first reviewer searched within the reference lists of obtained articles for other potentially relevant studies that were not retrieved by the electronic search.

Study selection

Types of Studies

The first reviewer screened the retrieved reports for eligibility through title, abstract, and full-text screening. The second reviewer checked the retrieved studies, and discrepancies were solved through discussion with a third reviewer. This meta-analysis included four randomized controlled studies and a case-control study that were published from inception to November 23, 2024.

Participants

Eligible studies included all studies that compared GC versus PC in CVDs. No restrictions were made with regard to age, sex, or race of the participants.

Interventions

Studies that were considered eligible included those that compared patients who received GC versus PC in CVDs.

Data extraction

The first reviewer carried out data extraction from the included studies using a standardized data sheet which included the following: (a) the study’s characteristics (author, year, country, study design); (b) patients’ characteristics (age at the time of treatment, sex, sample size); (c) intervention details (type, adverse effects, and the duration of follow up), and (e) the outcomes; primary outcomes: success rate in the form of clinical improvement which was standardized as disappearance or marked reduction of leg heaviness or fatigue, assessed by validated scoring tools, interface pressure, ejection fraction; secondary outcomes: tightness of leg, ease of donning. The second reviewer checked the collected data for consistency and clarity. Any disagreements were settled by referring to the third reviewer.

Measured outcomes

The primary outcomes of this study included the success rate, defined as the disappearance or significant improvement in the sensation of fatigue or heaviness, which was analyzed using odds ratios (ORs). In addition, objective measures such as interface pressure and improvement in ejection fraction were assessed using mean difference (MD). The secondary outcomes included patient-reported measures such as the sensation of leg tightness and the ease of donning, which were evaluated using ORs.

Assessment of the risk of bias in included studies

The NICE checklist for RCTs was used to assess risk of bias across randomization, blinding, allocation concealment, and outcome reporting [7]. The following domains were assessed: generation of random sequences, concealment of allocation, blinding of participants and assessors, outcome data completeness, and selective reporting. All the studies were rated as low, moderate, or high risk of bias across domains. Randomization and allocation concealment were adequate in all the studies. Blinding in Mosti and Partsch [3] was indefinite, which indicates possible performance bias. There was no selective reporting or attrition bias.

Data synthesis

**Figure 1:**
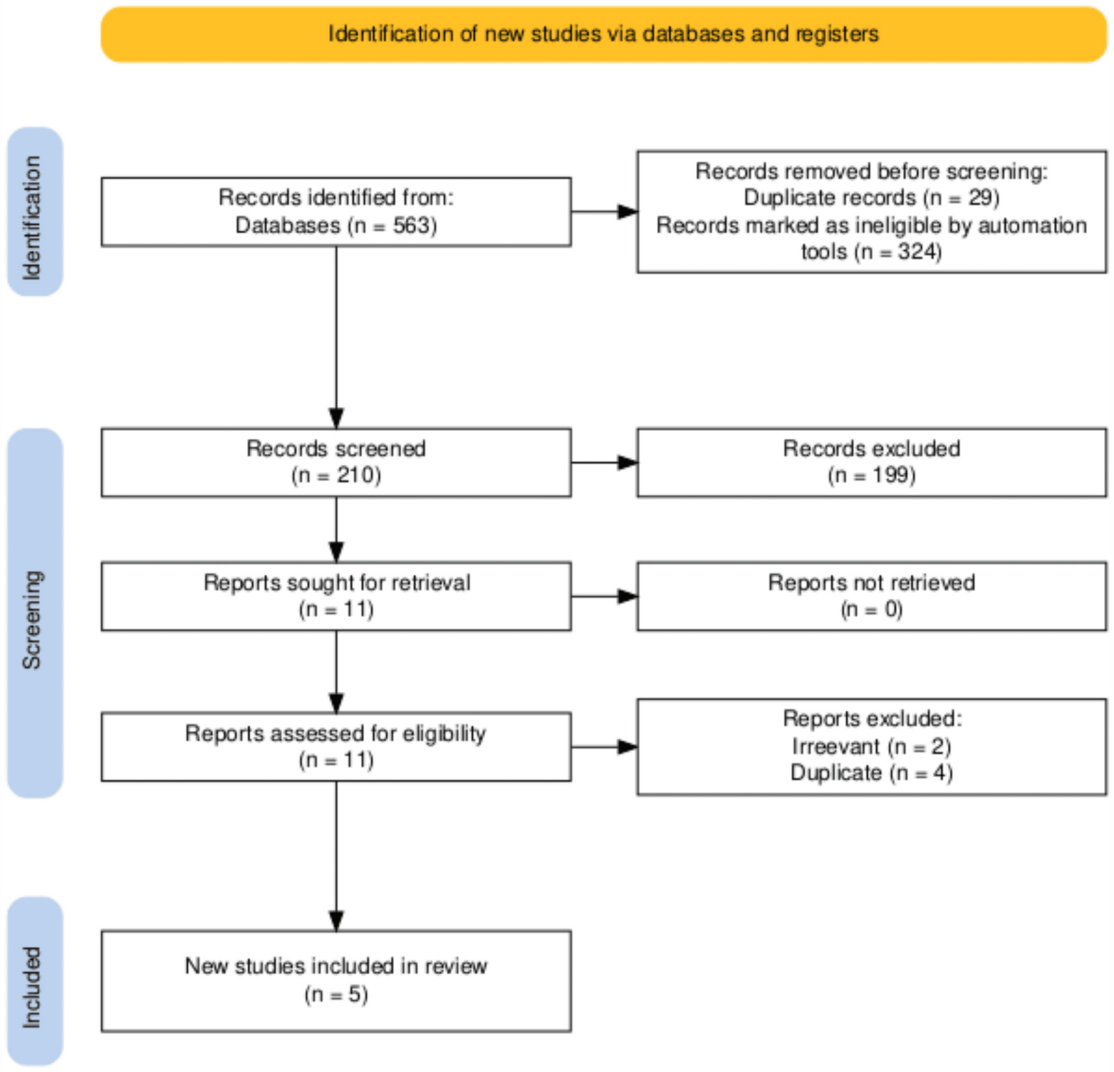
Preferred Reporting Items for Systematic Reviews and Meta-Analyses (PRISMA) flow chart

Initially, 563 records were retrieved from electronic database searches (Appendix A). After removing duplicates and excluded studies, 11 studies were finally eligible, of which five studies (874 individuals “patients and controls”) [3-5,8,9], were included (Table 1), while the six excluded studies from the MA were either irrelevant (n = 2) or duplicate (n = 4), these studies were mentioned in Figure 1 [10].

**Table 1:** Summary table of the included studies F: female, NM: not mentioned

Author	Year	Country	Study design	Age (mean± SD) or median (range)		Sex, no. (%)		Sample size		Primary outcomes	Secondary outcomes	Follow-up duration
				PECS	GECS	PECS	GECS	PECS	GECS			
Mosti and Partsch [3]	2011	Italy	RCT	57.2 years	57.2 years	18 F (60%)	18 F (60%)	30 patients	30 patients	Improvement in ejection fraction (EF) IFP exerted by graduated elastic compression stockings (GECS) and progressive elastic compression stockings (PECS).	Venous pumping function. Pressure gradients and dynamic changes during movement (e.g., tiptoe exercise).	NM
Couzan et al. [4]	2012	France	RCT-double blinded	53 (43-62)	54 (42-62)	F, 145 (72.8%)	F, 159 (78.7%)	199	202	Success rate (at three months)	Treatment success over time. Thromboembolic events. Compliance (at month 3). Ease application. Discomfort/harms/serious side effects.	Six months
Riebe et al. [5]	2016	Germany	Prospective, mono-centric, randomized controlled, cross-over study, double-blinded	Healthy volunteers: 26 (21-50), patients: 49.5 (23-81)	Healthy volunteers: 26 (21-50), patients: 49.5 (23-81)	Healthy volunteers: 19 F (59.3%), patients: 26 F (81.2%)	Healthy volunteers: 19 F (59.3%), patients: 26 F (81.2%)	64 (32 healthy volunteers, 32 CVI patients)		IFP. Ejection fraction. Venous filling index.	Comfort assessment (strangling, tightness, slipping tendency, ease of donning)	Seven days per stocking type, with a. One-week break between wearing periods.
Couzan et al. [8]	2009	France	RCT-double blinded	42.1 ± 13.0	42.6 ± 12.3	F, 43 (67.2%)	F, 46 (69.7%)	63	64	Disappearance or significant improvement in the sensation of fatigue or heaviness	Improvement in fatigue or leg heaviness. Ease of putting on the socks. The absence of discomfort.	After 15 days of wearing the socks
Mestre et al. [9]	2022	France	Case-control double-blinded	Controls (63.5 (53.070.0)), patients (61.0 (44.0-72.0))	Controls (61.0 (52.3-67.0)), patients (66.0 (60.0-76.5))	Patients (F, 41, 71.9%)-controls (F, 36, 66.6%)	Patients (F, 41, 71.9%)-controls (F, 36, 66.6%)	57 patients with CVD and 54 controls	57 patients with CVD and 54 controls	Interface pressure (IFP) changes. Vein cross-sectional area (SSV and DCV). Viscoelasticity.	Hysteresis loop variables (e.g., S2H). Dynamic pressure changes during postural adjustments.	Acute effects only

Statistical analysis

Meta-analysis was performed using Review Manager (RevMan) 5.4 version (The Cochrane Collaboration, Oxford, UK) [11]. For continuous variables, pooled MDs with 95% confidence intervals (CIs) were calculated. For dichotomous outcomes, pooled ORs with 95% CIs were computed. Statistical heterogeneity among studies was assessed using both the chi-squared (Cochran’s Q) test and the I² statistic. p < 0.10 on the chi-squared test or I² > 50% was considered indicative of significant heterogeneity. A random-effects model (DerSimonian-Laird) was chosen a priori to account for expected variability among trials. Sensitivity analyses were performed using a leave-one-out approach, excluding one study at a time to assess the robustness of pooled estimates. Statistical significance was defined as p < 0.05.

Results

Out of 563 studies, five studies with a total number of 874 patients and controls were eligible for inclusion in this meta-analysis comparing the patients who received GC versus PC in CVDs [3-5,8,9].

Primary outcomes

Clinical Improvement

**Figure 2:**
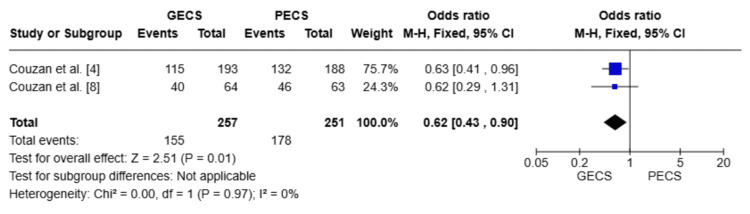
Forest plot comparing clinical improvement

Two studies with a total of 508 patients [4,8]. The PECS group demonstrated a statistically significant clinical improvement rate (OR = 0.62; 95% CI: (0.43-0.90), p = 0.01). The degree of heterogeneity between the pooled studies was negligible (I² = 0%), indicating consistency in the findings across studies (Figure 2).

Interface pressure (mmHg)

Interface Pressure at Level B1 (mmHg)

**Figure 3:**
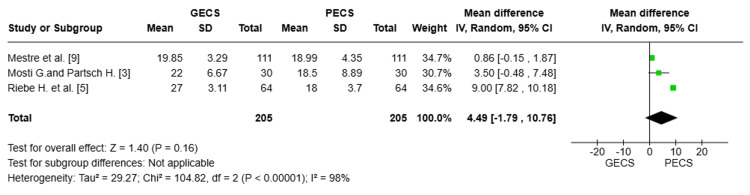
Forest plot comparing interface pressure (mmHg)at level B1

Data from three studies [9,3,5], with a total of 410 patients, were analyzed. The pooled MD (MD = 4.49; 95% CI: [-1.79 to 10.76], P = 0.16) indicated no statistically significant difference between the GECS and PECS groups in interface pressure at level B1. There was substantial heterogeneity among the studies (I² = 98%), suggesting considerable variability in the results across the included studies. This heterogeneity was best resolved by the exclusion of Riebe et al. (Figure 3) [5].

Interface Pressure at Level C (mmHg)

**Figure 4:**
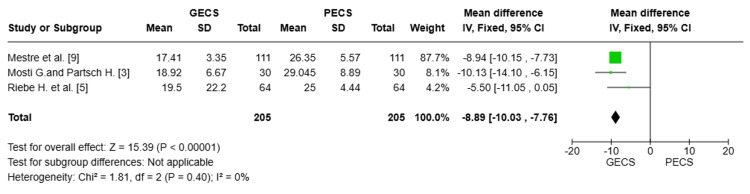
Forest plot comparing interface pressure (mmHg) at level C

Three studies [3,5,9], with a total of 410 patients, were analyzed. The PECS group demonstrated a statistically significant elevation in interface pressure compared to the GECS group at level C (MD= -8.89 mmHg; 95% CI: (-10.03 to -7.76), p < 0.00001. This indicates stronger compression at the calf level with PECS, a physiologically critical zone for venous return. There was no heterogeneity among the pooled studies (I² = 0%), indicating consistent findings across the included studies (Figure 4).

Improvement in ejection fraction

**Figure 5:**
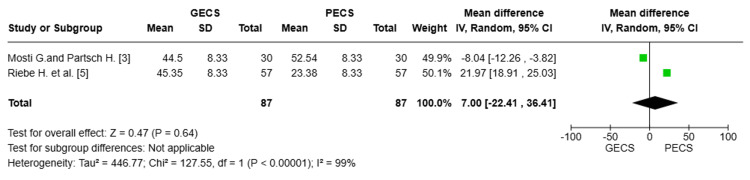
Forest plot comparing the improvement in ejection fraction

Two studies with a total of 174 patients were pooled [3,5]. There was no statistically significant difference between the GECS and PECS groups regarding the improvement in ejection fraction (MD = 7.00; 95% CI: (-22.41, 36.41), p = 0.64). The pooled studies exhibit substantial heterogeneity with I² = 99%, which could not be resolved (Figure 5).

Secondary outcomes

Tightness of Leg Sensation

**Figure 6:**
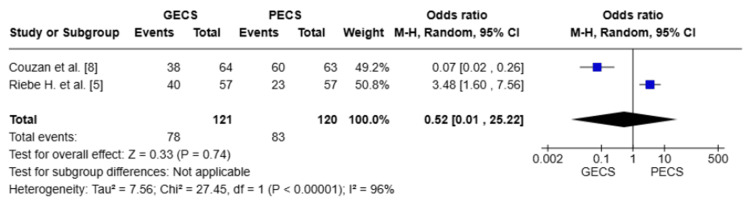
Forest plot regarding the tightness of leg

Two studies with a total of 241 patients [5,8]. There was no statistically significant difference between the GECS and PECS groups in terms of leg tightness (OR = 0.52; 95% CI: (0.01-25.22), p = 0.74). The pooled studies show substantial heterogeneity with I² = 96%, which could not be resolved (Figure 6).

Ease of donning

**Figure 7:**
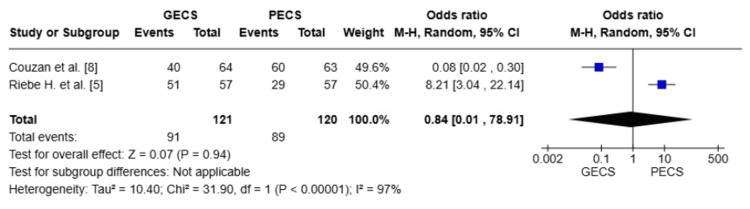
Forest plot comparing ease of donning

Pooled data from two studies (n = 241) [5,8] showed no statistically significant difference between the GECS and PECS groups regarding the ease of donning (OR = 0.84; 95% CI: (0.01-78.91), p = 0.94). The pooled studies exhibit substantial heterogeneity with I² = 97%, which could not be resolved (Figure 7).

Risk of bias

The risk of bias assessment for the included studies revealed that all studies demonstrated adequate randomization sequence generation and allocation concealment; however, potential concerns arise in other areas. Blinding of participants and personnel, as well as blinding of outcome assessment, were generally adequately addressed, except for Mosti and Partsch [3], raising the possibility of performance and detection bias. Incomplete outcome data and selective reporting were also adequately documented, excluding attrition and reporting bias.

**Figure 8:**
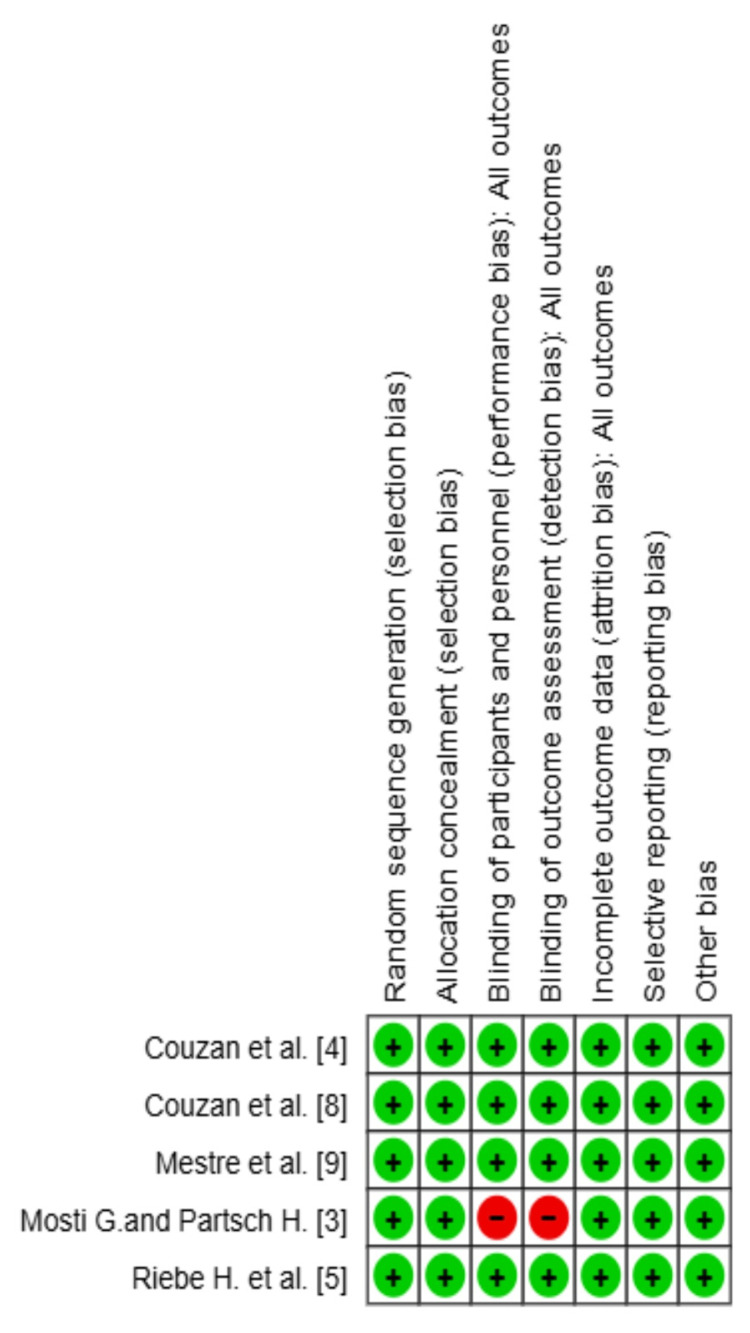
Cochrane risk of bias summary References: [3-5,8,9]

**Figure 9:**
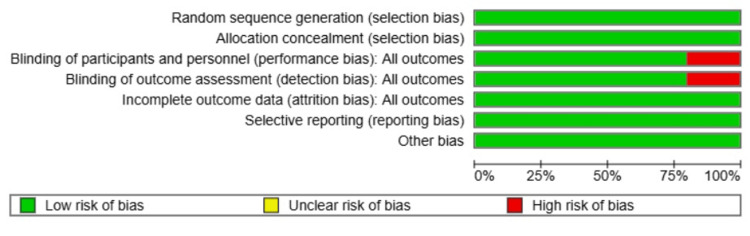
Cochrane risk of bias graph References: [3-5,8,9]

Overall, the ROB assessment suggests that while the included studies generally adhered to good methodological practices in most aspects and ROB domains, there were notable areas where further attention is needed to minimize potential biases (Figures 8-9).

Discussion

CVI results from impaired venous return and elevated venous pressure, leading to pain, edema, and ulceration [12]. Compression therapy remains the first-line treatment due to its ability to reduce venous hypertension and improve microcirculation [13].

Two common types of compression therapy are GC (GECS) and PC (PECS) [12]. GECS involves applying graduated pressure to the legs, with higher pressure at the ankle and gradually decreasing pressure towards the thigh. While GECS is effective in reducing symptoms, it can have some limitations, such as discomfort, difficulty in donning and doffing, and potential skin irritation [13].

PECS, on the other hand, delivers incrementally increasing compression toward the calf, enhancing venous ejection fraction and microcirculatory flow. PECS offers several advantages over GECS, including improved comfort, easier application, and enhanced therapeutic efficacy [5].

This meta-analysis included five studies involving 874 patients to compare the efficacy and safety of GECS and PECS in patients with CVDs. While the findings provide preliminary data on the relative efficacy of these modalities, the small number of studies and the heterogeneity observed in several outcomes require cautious interpretation [14,15].

Interface pressure analysis revealed a differential effect by anatomical level. Comparison of the two groups at level B1 did not show a significant difference, and high heterogeneity suggested study design and measurement variability. At the calf level, higher interface pressure with PECS aligns with previous hemodynamic evidence that effective venous outflow depends on adequate calf compression rather than ankle compression alone. This may explain improved subjective outcomes among PECS users. Although this difference does not directly confirm improved clinical outcomes, it might be physiologically relevant since maintaining adequate pressure at the calf level has been proven by earlier hemodynamic studies to improve venous return and reduce stasis.

Although ejection fraction and comfort outcomes did not differ significantly, wide heterogeneity indicates inconsistencies in study design and outcome measurement. Standardized assessment protocols and validated patient-reported measures are necessary in future trials [16]. Similarly, for secondary outcomes such as perceived leg tightness and ease of donning, no statistically significant differences were detected. However, high heterogeneity across studies limits the strength of these findings, and larger, more standardized trials are required to clarify whether meaningful differences exist in patient comfort and adherence [17].

The overall risk of bias assessment was generally acceptable, with most studies demonstrating appropriate randomization and allocation concealment. Nonetheless, concerns were noted in areas such as blinding and outcome reporting, particularly in older trials. These methodological limitations should be considered when interpreting the results, as they may have introduced performance and detection bias.

The findings of this review align with prior reports that have emphasized the challenges associated with GECS, including difficulties in donning, reduced patient compliance, and discomfort due to excessive pressure at certain levels of the limb [18,19]. PECS, with its more tailored pressure distribution, appears to overcome some of these limitations, offering greater clinical efficacy and potentially improving patient adherence [20,21].

Finally, the findings are consistent with newer investigations emphasizing personalized compression profiles and digitally calibrated stocking pressure systems, which may improve adherence and long-term vascular outcomes [15]. While the evidence is encouraging, the relatively small sample sizes, heterogeneity in secondary outcomes, and the limited number of trials underscore the need for further research.

Limitations

This meta-analysis has several limitations. First, the evidence base is limited to five studies with moderate sample sizes, reducing statistical power and generalizability. Second, high heterogeneity in some outcomes-particularly interface pressure at level B1 and ejection fraction-reflects variability in study protocols, patient severity, and measurement techniques. Third, despite a generally low risk of bias, incomplete blinding in some trials may have introduced performance bias. Fourth, variations in follow-up duration and outcome definitions may affect comparability. Finally, long-term effects, patient compliance, and cost-effectiveness were not addressed. Therefore, conclusions should be interpreted with caution but remain hypothesis-generating for future high-quality RCTs.

## Conclusions

This meta-analysis of five studies (874 participants) suggests that PC (PECS) provides superior clinical improvement compared with GC (GECS) (OR = 0.62; 95% CI: 0.43-0.90; p = 0.01; I² = 0%), and exerts higher interface pressure at the calf level (MD = -8.89 mmHg; 95% CI: -10.03 to -7.76; p < 0.00001; I² = 0%). No significant differences were found in ejection fraction, leg tightness, or ease of donning.

While these results indicate a possible advantage of PECS in clinical improvement and calf-level interface pressure, the small number of included studies and substantial heterogeneity limit the strength of these conclusions. Further high-quality, larger randomized trials are warranted to validate these findings.

## Appendices

Appendix A 

**Table 2:** Search terms used for each database and the count of search results

Search terms	Library	Number of retrieved articles		Number of articles	Causes for exclusion
(chronic venous insufficiency) AND (graduated compression stockings OR GECS) AND (progressive compression stockings OR negative compression stockings OR degressive compression stockings OR PECS)	PubMed	13	Included after removal of duplications	534	Automatic duplicate removal
("chronic venous disease" AND "graduated compression" AND "progressive compression")	Science direct	518	Included after other exclusions	210	Review articles
('chronic venous insufficiency'/exp OR 'chronic venous disease') AND ('graduated compression stockings' OR GECS) AND ('progressive compression stockings' OR 'degressive compression stockings' OR PECS)	WOS	14	Included after abstract screening	11	-
TITLE-ABS-KEY("chronic venous insufficiency" OR "chronic venous disease") AND TITLE-ABS-KEY("graduated compression stockings" OR GECS) AND TITLE-ABS-KEY("progressive compression stockings" OR "degressive compression stockings" OR PECS)	Scopus	9	Included after full text screening, i.e., to extraction	5	Irrelevant (n = 2) or duplicate (n = 4)
(chronic venous insufficiency OR chronic venous disease) AND (graduated compression OR progressive compression)	CENTRAL	7	-	-	-
Manual		2	-	-	-
Total		563	-	-	-
